# A Further Case for Targeting PRMT5 and the ERK1/2 and PI3K Pathways in CRC

**DOI:** 10.3390/ijms262110416

**Published:** 2025-10-27

**Authors:** Mark Spivak, Moshe Pahmer, Dorna Delrahimnia, Tzuriel Sapir, David Shifteh

**Affiliations:** 1 College of Medicine, SUNY Downstate Health Sciences University, Brooklyn, NY 11203, USA; mark.spivak@downstate.edu; 2 Department of Biology, Yeshiva College, Yeshiva University, New York, NY 10033, USA; mpahmer@mail.yu.edu; 3 College of Health Professions, Pace University, New York, NY 10038, USA; dd46464n@pace.edu; 4 Perelman School of Medicine, University of Pennsylvania, Philadelphia, PA 19104, USA

**Keywords:** PRMT5, CRC, KRAS, RAF, MEK, ERK, PI3K, AKT, mTOR, PTEN

## Abstract

Colorectal cancer (CRC) is the second leading cause of cancer-related mortality in the United States. Recent breakthroughs in research are highlighting the complex genetic and epigenetic alterations driving CRC progression. Among these, the ERK1/2 and PI3K pathways are central regulators of cellular proliferation, survival, and differentiation. The overactivation of these pathways is frequently observed in cancer and is associated with poor patient prognosis. Protein Arginine Methyltransferase 5 (PRMT5), a key epigenetic regulator, has been implicated in modulating the ERK1/2 and PI3K pathways in cancer. Previous studies, including those from our own group, are starting to suggest that targeting PRMT5 and the ERK1/2 and PI3K pathways may offer therapeutic benefits. Thus, we sought to provide further evidence of the relationship between PRMT5 and the ERK1/2 and PI3K pathways in CRC. Using patient tumor gene expression data and protein–protein interaction networks, we provide further evidence that PRMT5 is positively correlated with, and interacts with, the ERK1/2 and PI3K pathways in CRC. These findings are significant, as they further strengthen the case for the urgent need of additional research into therapeutic strategies targeting PRMT5 and the ERK1/2 and PI3K pathways in CRC.

## 1. Introduction

Colorectal cancer (CRC) is a significant public health challenge, ranking second in mortality among all cancers affecting men and women in the United States [1]. Despite advancements in early detection and treatment, survival outcomes for metastatic CRC remain poor, with fewer than 20% of patients surviving beyond five years after diagnosis [2]. However, recent breakthroughs in research are shedding light on the diverse genetic and epigenetic alterations underlying CRC progression [3,4,5,6,7,8,9].

The ERK1/2 and PI3K pathways are the chief signaling pathways for cellular proliferation, survival, and differentiation [5]. The overactivation of the ERK1/2 and PI3K pathways is commonly seen in cancer and is associated with poor patient prognosis [10]. Increasing evidence is showcasing that epigenetic alterations play a major role in regulating the ERK1/2 and PI3K pathways [11,12,13].

Protein Arginine Methyltransferase 5 (PRMT5) is an epigenetic regulator currently undergoing clinical trials as a potential therapeutic target for cancer [14]. PRMT5 has been shown to be overexpressed and negatively correlated with patient survival in many types of cancers [15,16,17,18]. Recent studies indicate that PRMT5 is involved in regulating the ERK1/2 and PI3K pathways [10]. PRMT5 has been shown to interact with many components of the ERK1/2 and PI3K pathways, including EGFR, Fibroblast Growth Factor Receptor 3 (FGFR3), RAF, ERK, PTEN, AKT, and more [10,19,20,21]. PRMT5 exerts differential regulatory effects on upstream and downstream nodes within the PI3K and MAPK signaling pathways. PRMT5 primarily modulates downstream effectors such as AKT and mTOR through direct methylation events. In contrast, PRMT5’s influence on upstream nodes is more indirect, leading to their enhanced degradation and reduced catalytic activity. This results in a dampening of the ERK1/2 signal amplitude [4,10,22].

Of particular interest, previous work from our group has indicated that PRMT5 interacts with KRAS, an upstream activator of both the ERK1/2 and PI3K pathways [23,24]. KRAS mutations are found in nearly 45% of CRCs and act as a molecular switch to constitutively stimulate cellular growth, survival, and proliferation, leading to tumorigenesis [25]. Yet, despite decades of intense research, a selective inhibitor for KRAS is yet to be developed. Patients with KRAS-mutant CRC, therefore, experience poor outcomes and a worse prognosis [26]. Our group’s previous work showed that KRAS-mutant CRC cells showed a greater therapeutic response to PRMT5 inhibition treatment compared to KRAS-WT CRC cells [23]. As such, our group’s work, as well as other studies, are starting to demonstrate that targeting PRMT5 and the ERK1/2 and PI3K pathways may offer therapeutic benefits [27,28,29,30].

In fact, recent clinical progress with PRMT5 inhibitors such as GSK3326595 and JNJ-64619178 has highlighted their therapeutic potential in molecularly defined subgroups of CRC [31,32,33]. Predictive biomarkers such as PRMT5 expression, MTAP deletion, and APC loss confer an increased dependency on PRMT5 and a greater sensitivity to its inhibition [34,35]. MTAP-deleted CRCs are especially vulnerable to MTA-cooperative PRMT5 inhibitors, and combinatorial screening has revealed a strong synergy between PRMT5 blockade and the inhibition of MAPK components including KRAS, MEK, ERK, and RAF (26078354). Moreover, dual targeting of PRMT5 and the PI3K/mTOR or MEK/ERK pathways enhances anti-tumor efficacy, supporting the development of biomarker-guided combination strategies to improve therapeutic outcomes in CRC [36,37,38]. Thus, we sought to provide further evidence of the relationship between PRMT5 and the ERK1/2 and PI3K pathways in CRC. Using patient tumor gene expression data and protein–protein interaction networks, we provide further evidence that PRMT5 is positively correlated with, and interacts with, the ERK1/2 and PI3K pathways in CRC. These findings are significant, as they further strengthen the case for the urgent need of additional research into therapeutic strategies targeting PRMT5 and the ERK1/2 and PI3K pathways in CRC.

## 2. Results

### 2.1. The ERK1/2 and PI3K Pathway Proteins Are Positively Correlated with PRMT5 in Colon and Rectum Patient Tumor Samples

**Figure 1:**
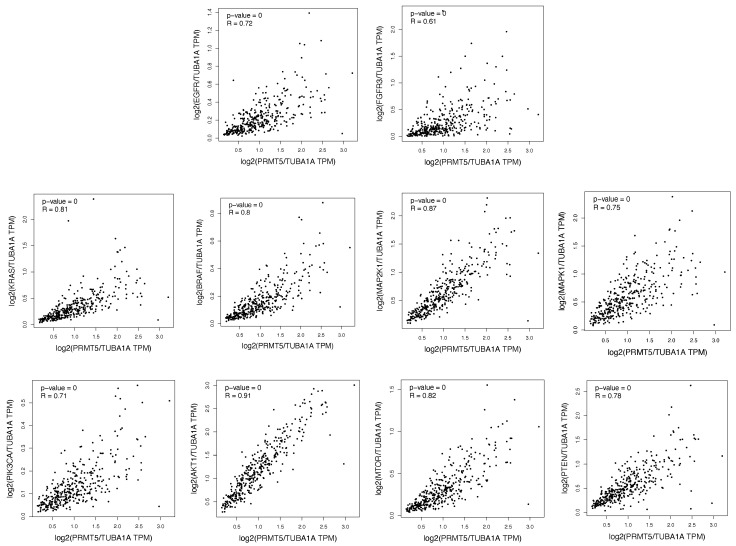
The ERK1/2 and PI3K pathway proteins are shown to be positively correlated with PRMT5 in colon and rectum patient tumor samples (*R*-Value > 0.61, *p*-Value < 0.01).

Previous studies, including those from our own group, are starting to suggest that targeting PRMT5 and the ERK1/2 and PI3K pathways may offer therapeutic benefits. Thus, we sought to provide further evidence of the relationship between PRMT5 and the ERK1/2 and PI3K pathways in CRC. As such, we first used the GEPIA database to analyze colon and rectum patient tumor gene expression data to determine whether the ERK1/2 and PI3K pathway proteins have an expressional correlation with PRMT5 in colon and rectum patient tumor samples. Our results show that the ERK1/2 and PI3K pathway proteins are positively correlated with PRMT5 with a *R*-Value > 0.61 and a *p*-Value < 0.01. The analyzed colon and rectum patient tumor gene expression data plots for the ERK1/2 and PI3K pathway proteins can be seen in Figure 1 below.

### 2.2. The ERK1/2 and PI3K Pathway Proteins Have Demonstrated Interactations with PRMT5 in the STRING Protein–Protein Interaction Network

**Figure 2:**
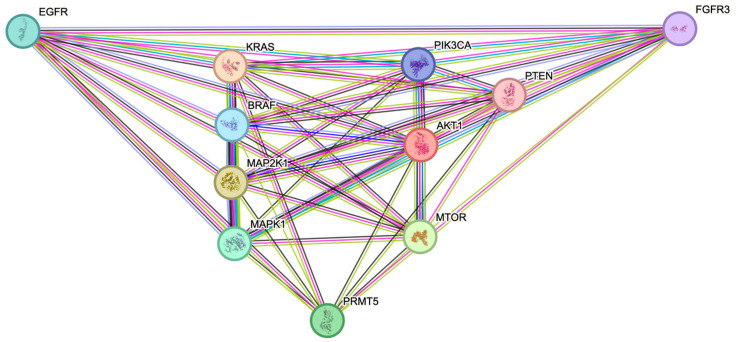
The receptor proteins EGFR and FGFR3, the ERK1/2 pathway proteins KRAS, BRAF, MEK (MAP2K1), and ERK (MAPK1), and lastly the PI3K pathway proteins AKT (AKT1), mTOR, and PTEN are shown to interact with PRMT5 in the STRING database (Interaction score > 0.150).

After identifying that the ERK1/2 and PI3K pathway proteins have an expressional correlation with PRMT5 in colon and rectum patient tumor samples, we next used the STRING database to determine which of the ERK1/2 and PI3K pathway proteins have demonstrated interactions with PRMT5. Our results show that PRMT5 interacts with the receptor proteins EGFR and FGFR3, the ERK1/2 pathway proteins KRAS, RAF, MEK, and ERK, and lastly the PI3K pathway proteins AKT, mTOR, and PTEN, with an interaction score > 0.150. The analyzed protein–protein interaction network for the ERK1/2 and PI3K pathway proteins can be seen in Figure 2 below.

### 2.3. Protein Network Map and Tabular Model Illustrates the Proteins in the ERK1/2 and PI3K Pathways That Both Positively Correlate with, as Well as Interact with, PRMT5

**Figure 3:**
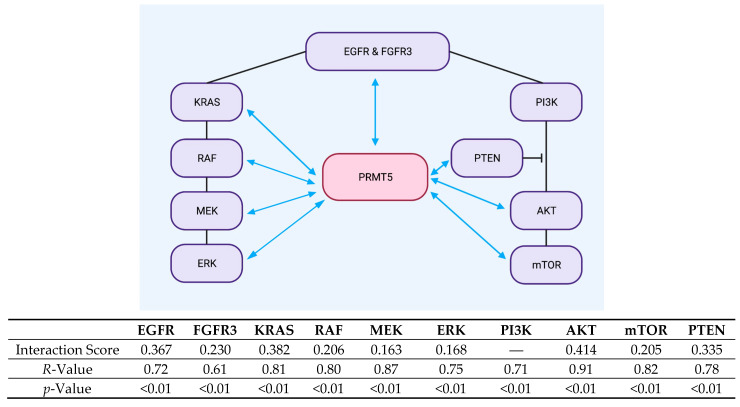
Protein network map and tabular model are shown, which illustrate that the following ERK1/2 and PI3K pathway proteins, EGFR, FGFR3, KRAS, RAF, MEK, ERK, AKT, mTOR, and PTEN, both positively correlate with (*R*-Value > 0.61, *p*-Value < 0.01) and interact with (interaction score > 0.150) PRMT5.

After identifying that the receptor proteins EGFR and FGFR3, the ERK1/2 pathway proteins KRAS, RAF, MEK, and ERK, and lastly the PI3K pathway proteins AKT, mTOR, and PTEN have demonstrated interactions with PRMT5 in the STRING database, we next created a tabular model and used Biorender to develop a protein network map to illustrate the proteins in the ERK1/2 and PI3K pathways that both positively correlate with, as well as interact with, PRMT5. Our protein network map and tabular model demonstrate that PRMT5 is positively correlated with (*R*-Value > 0.61, *p*-Value < 0.01) and interacts with (interaction score > 0.150) the following ERK1/2 and PI3K pathway proteins: EGFR, FGFR3, KRAS, RAF, MEK, ERK, AKT, mTOR, and PTEN. The analyzed protein network map and tabular model for the ERK1/2 and PI3K pathway proteins can be seen in Figure 3 below.

## 3. Discussion

CRC remains a major public health concern, ranking as the second leading cause of cancer-related death among men and women in the United States. Emerging research is continuing to uncover that complex genetic and epigenetic alterations are driving CRC progression, which provides new insights into potential therapeutic targets.

The ERK1/2 and PI3K pathways serve as central signaling cascades governing cellular proliferation, survival, and differentiation. Aberrant activation of these pathways is frequently observed in cancer and is strongly linked to unfavorable patient outcomes. Growing evidence highlights the significant role of epigenetic mechanisms in modulating ERK1/2 and PI3K signaling.

Protein Arginine Methyltransferase 5 (PRMT5), an epigenetic enzyme currently under investigation in clinical trials, has emerged as a promising therapeutic target in oncology. PRMT5 overexpression has been reported across numerous cancer types and is often associated with reduced patient survival. Recent findings suggest that PRMT5 can influence both the ERK1/2 and PI3K pathways. Furthermore, our group and others have demonstrated that co-targeting PRMT5 alongside these signaling networks may provide enhanced therapeutic benefit.

Thus, we sought to provide further evidence of the relationship between PRMT5 and the ERK1/2 and PI3K pathways in CRC. As such, we first used the GEPIA database to analyze colon and rectum patient tumor gene expression data to determine whether the ERK1/2 and PI3K pathway proteins have an expressional correlation with PRMT5 in colon and rectum patient tumor samples. Our results show that the ERK1/2 and PI3K pathway proteins are positively correlated with PRMT5, with a *R*-Value > 0.61 and a *p*-Value < 0.01.

We next used the STRING database to determine which of the ERK1/2 and PI3K pathway proteins have demonstrated interactions with PRMT5. Our results show that PRMT5 interacts with the receptor proteins EGFR and FGFR3, the ERK1/2 pathway proteins KRAS, RAF, MEK, and ERK, and lastly the PI3K pathway proteins AKT, mTOR, and PTEN, with an interaction score > 0.150.

Finally, we used Biorender to develop a protein network map to model the proteins in the ERK1/2 and PI3K pathways that both positively correlate with, as well as interact with, PRMT5. Our protein network map demonstrates that PRMT5 is positively correlated with (*R*-Value > 0.61, *p*-Value < 0.01) and interacts with (interaction score > 0.150) the following ERK1/2 and PI3K pathway proteins: EGFR, FGFR3, KRAS, RAF, MEK, ERK, AKT, mTOR, and PTEN.

Our study thus shows further evidence that PRMT5 is positively correlated with, and interacts with, the ERK1/2 and PI3K pathways in CRC. These findings are significant, as they further strengthen the case for the urgent need of additional research into therapeutic strategies targeting PRMT5 and the ERK1/2 and PI3K pathways in CRC. It is important to note that, although our correlation analysis in Figure 1 highlights a strong association between PRMT5 and multiple nodes of the ERK1/2 and PI3K pathways, it does not prove causation or direct molecular interaction and does not show a linear regression correlation curve either. Furthermore, the interaction scores between PRMT5 and several of the ERK1/2 and PI3K pathway proteins are on the lower end, which makes the likelihood of an indiscriminate interaction between PRMT5 and all of the ERK1/2 and PI3K pathway proteins less likely. As such, additional research is urgently needed to delineate the exact molecular mechanisms behind PRMT5’s interactions with the ERK1/2 and PI3K pathways, as well as the specificity of these findings in KRAS-mutant CRC, as this can aid in the development of much needed therapeutic strategies to target PRMT5 and the ERK1/2 and PI3K pathways in CRC.

## 4. Materials and Methods

### 4.1. Gene Expression Profiling Interactive Analysis (GEPIA)

The GEPIA database (Peking University, Beijing, China) was used to analyze the RNA-Seq data of colon and rectum cancer patients from The Cancer Genome Atlas (TCGA) database to determine which of the ERK1/2 and PI3K pathway proteins are positively correlated with PRMT5 in colon and rectum patient tumor samples [39]. This was performed by using the GEPIA database’s Multiple Gene Analysis and Correlation Analysis. The generated data were normalized with the TUBA1A gene, and the Spearman Correlation Coefficient was determined. The results of the colon and rectum patient tumor gene expression were then analyzed.

### 4.2. STRING Protein–Protein Interaction Network

The STRING protein–protein interaction network (European Molecular Biology Laboratory (EMBL), Heidelberg, Germany) was used to determine which of the ERK1/2 and PI3K pathway proteins have demonstrated interactions with PRMT5 [40]. This was performed by using the STRING database, selecting multiple proteins, and listing all of the determined protein names and Homo sapiens for the organism. The results of the protein–protein interaction network were then analyzed.

### 4.3. Biorender: Scientific Image and Illustration Software

The Biorender Scientific Image and Illustration Software (BioRender, Toronto, ON, Canada) (https://www.biorender.com) was used to generate a protein network map that models the proteins in the ERK1/2 and PI3K pathways that both positively correlate with, as well as interact with, PRMT5. This was performed by using the Biorender Scientific Image and Illustration Software and developing the protein network map that models the proteins in the ERK1/2 and PI3K pathways that both positively correlate with, as well as interact with, PRMT5. The results of the protein network map were then analyzed.

## 5. Conclusions

In conclusion, previous studies, including those from our own group, are starting to suggest that targeting PRMT5 and the ERK1/2 and PI3K pathways may offer therapeutic benefits. Thus, we sought to provide further evidence of the relationship between PRMT5 and the ERK1/2 and PI3K pathways in CRC. Using patient tumor gene expression data and protein–protein interaction networks, we provide further evidence that PRMT5 is positively correlated with, and interacts with, the ERK1/2 and PI3K pathways in CRC. These findings are significant, as they further strengthen the case for the urgent need of additional research into therapeutic strategies targeting PRMT5 and the ERK1/2 and PI3K pathways in CRC.

## Data Availability

The raw data presented and analyzed in this study can be obtained from the GEPIA and STRING databases as explained in the Materials and Methods Section 4 above.

## Abbreviations

The following abbreviations are used in this manuscript:

PRMT5	Protein Arginine Methyltransferase 5
CRC	Colorectal cancer
ERK1/2	Extracellular signal-regulated kinases 1 and 2
KRAS	Kirsten rat sarcoma viral oncogene homolog
GEPIA	Gene Expression Profiling Interactive Analysis
